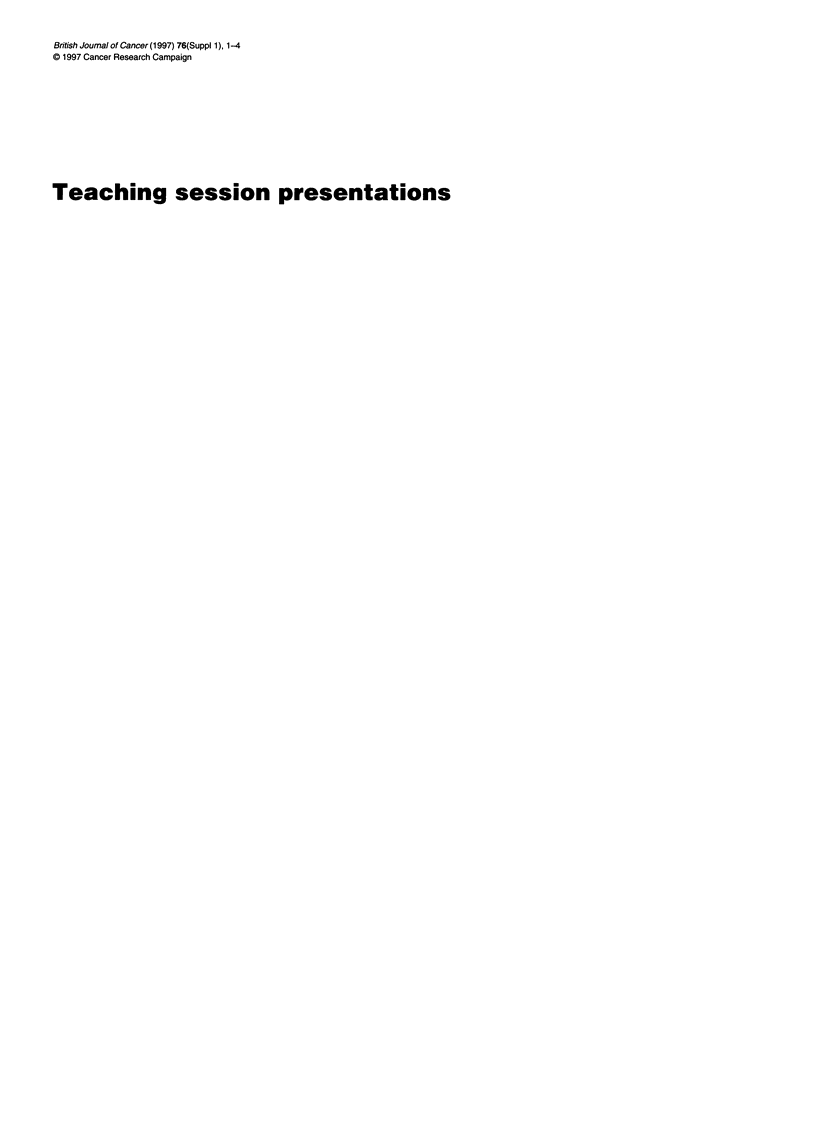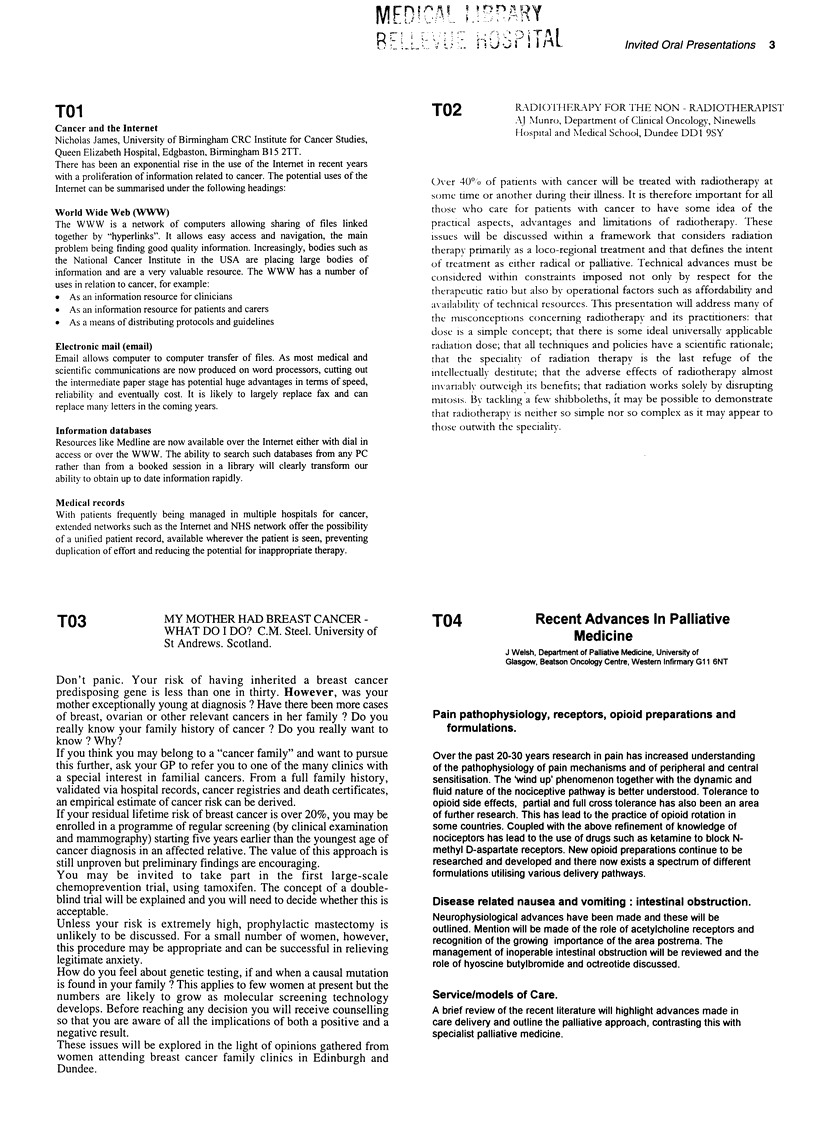# Joint meeting of the British Oncological Association, Association of Cancer Physicians, and Royal College of Radiologists. United Kingdom, 5-8 July 1997. Abstracts.

**Published:** 1997

**Authors:** 


*British Journal of Cancer* (1997) **76**(Suppl 1), 1–4
© 1997 Cancer Research Campaign

### Teaching session presentations

MEDICAL LIBRARY
BELLEVUE HOSPITAL

### T01

**Cancer and the Internet**

Nicholas James, University of Birmingham CRC Institute for Cancer Studies, Queen Elizabeth Hospital, Edgbaston, Birmingham B15 2TT.

There has been an exponential rise in the use of the Internet in recent years with a proliferation of information related to cancer. The potential uses of the Internet can be summarised under the following headings:

**World Wide Web (WWW)**

The WWW is a network of computers allowing sharing of files linked together by "hyperlinks". It allows easy access and navigation, the main problem being finding good quality information. Increasingly, bodies such as the National Cancer Institute in the USA are placing large bodies of information and are a very valuable resource. The WWW has a number of uses in relation to cancer, for example:

- As an information resource for clinicians
- As an information resource for patients and carers
- As a means of distributing protocols and guidelines

**Electronic mail (email)**

Email allows computer to computer transfer of files. As most medical and scientific communications are now produced on word processors, cutting out the intermediate paper stage has potential huge advantages in terms of speed, reliability and eventually cost. It is likely to largely replace fax and can replace many letters in the coming years.

**Information databases**

Resources like Medline are now available over the Internet either with dial in access or over the WWW. The ability to search such databases from any PC rather than from a booked session in a library will clearly transform our ability to obtain up to date information rapidly.

**Medical records**

With patients frequently being managed in multiple hospitals for cancer, extended networks such as the Internet and NHS network offer the possibility of a unified patient record, available wherever the patient is seen, preventing duplication of effort and reducing the potential for inappropriate therapy.

### T02

RADIOTHERAPY FOR THE NON - RADIOTHERAPIST

AJ Munro, Department of Clinical Oncology, Ninewells Hospital and Medical School, Dundee DD1 9SY

Over 40% of patients with cancer will be treated with radiotherapy at some time or another during their illness. It is therefore important for all those who care for patients with cancer to have some idea of the practical aspects, advantages and limitations of radiotherapy. These issues will be discussed within a framework that considers radiation therapy primarily as a loco-regional treatment and that defines the intent of treatment as either radical or palliative. Technical advances must be considered within constraints imposed not only by respect for the therapeutic ratio but also by operational factors such as affordability and availability of technical resources. This presentation will address many of the misconceptions concerning radiotherapy and its practitioners: that dose is a simple concept; that there is some ideal universally applicable radiation dose; that all techniques and policies have a scientific rationale; that the speciality of radiation therapy is the last refuge of the intellectually destitute; that the adverse effects of radiotherapy almost invariably outweigh its benefits; that radiation works solely by disrupting mitosis. By tackling a few shibboleths, it may be possible to demonstrate that radiotherapy is neither so simple nor so complex as it may appear to those outwith the speciality.

### T03

MY MOTHER HAD BREAST CANCER - WHAT DO I DO? C.M. Steel. University of St Andrews. Scotland.

Don't panic. Your risk of having inherited a breast cancer predisposing gene is less than one in thirty. **However**, was your mother exceptionally young at diagnosis ? Have there been more cases of breast, ovarian or other relevant cancers in her family ? Do you really know your family history of cancer ? Do you really want to know ? Why?

If you think you may belong to a "cancer family" and want to pursue this further, ask your GP to refer you to one of the many clinics with a special interest in familial cancers. From a full family history, validated via hospital records, cancer registries and death certificates, an empirical estimate of cancer risk can be derived.

If your residual lifetime risk of breast cancer is over 20%, you may be enrolled in a programme of regular screening (by clinical examination and mammography) starting five years earlier than the youngest age of cancer diagnosis in an affected relative. The value of this approach is still unproven but preliminary findings are encouraging.

You may be invited to take part in the first large-scale chemoprevention trial, using tamoxifen. The concept of a double-blind trial will be explained and you will need to decide whether this is acceptable.

Unless your risk is extremely high, prophylactic mastectomy is unlikely to be discussed. For a small number of women, however, this procedure may be appropriate and can be successful in relieving legitimate anxiety.

How do you feel about genetic testing, if and when a causal mutation is found in your family ? This applies to few women at present but the numbers are likely to grow as molecular screening technology develops. Before reaching any decision you will receive counselling so that you are aware of all the implications of both a positive and a negative result.

These issues will be explored in the light of opinions gathered from women attending breast cancer family clinics in Edinburgh and Dundee.

### T04

**Recent Advances In Palliative Medicine**

J Welsh, Department of Palliative Medicine, University of Glasgow, Beatson Oncology Centre, Western Infirmary G11 6NT

### Pain pathophysiology, receptors, opioid preparations and formulations.

Over the past 20-30 years research in pain has increased understanding of the pathophysiology of pain mechanisms and of peripheral and central sensitisation. The 'wind up' phenomenon together with the dynamic and fluid nature of the nociceptive pathway is better understood. Tolerance to opioid side effects, partial and full cross tolerance has also been an area of further research. This has lead to the practice of opioid rotation in some countries. Coupled with the above refinement of knowledge of nociceptors has lead to the use of drugs such as ketamine to block N-methyl D-aspartate receptors. New opioid preparations continue to be researched and developed and there now exists a spectrum of different formulations utilising various delivery pathways.

### Disease related nausea and vomiting : intestinal obstruction.

Neurophysiological advances have been made and these will be outlined. Mention will be made of the role of acetylcholine receptors and recognition of the growing importance of the area postrema. The management of inoperable intestinal obstruction will be reviewed and the role of hyoscine butylbromide and octreotide discussed.

### Service/models of Care.

A brief review of the recent literature will highlight advances made in care delivery and outline the palliative approach, contrasting this with specialist palliative medicine.